# Tele-Buprenorphine Initiations for Opioid Use Disorder Without In-Person Relationships

**DOI:** 10.1001/jamanetworkopen.2025.0001

**Published:** 2025-03-03

**Authors:** Emma E. McGinty, Yimin Ge, Jiani Yu, Kayla N. Tormohlen, Matthew D. Eisenberg

**Affiliations:** 1 Division of Health Policy and Economics, Weill Cornell Medical College and the Cornell Health Policy Center, New York, New York; 2 Department of Health Policy and Management, Johns Hopkins Bloomberg School of Public Health, Baltimore, Maryland

## Abstract

This cross-sectional study examines the proportion of individuals with opioid use disorder (OUD) who initiated buprenorphine treatment without in-person visits under a temporary rule allowing tele-initiation of controlled substances.

## Introduction

In response to the COVID-19 pandemic, the US Drug Enforcement Administration (DEA) temporary telehealth rules waived the Ryan Haight Act requirement that clinicians examine a patient in person prior to prescribing a controlled substance.^1^ This March 16, 2020, change facilitated increased tele-initiation of buprenorphine, a lifesaving but underused medication for opioid use disorder (OUD).^2,3^ After the temporary telehealth rules expire on December 31, 2025,^4^ the DEA’s proposed rule for tele-prescribing thereafter requires an in-person visit with the buprenorphine-initiating clinician prior to or within 30 days of tele-initiation.^1^ To understand the proposed rule’s potential impact on buprenorphine initiations, we assessed the proportion of tele-initiations that did not meet these proposed in-person requirements from 2020 to 2022.

## Methods

In this cross-sectional study, we used IQVIA claims capturing services delivered by 75% of US licensed physicians.^5^ We created a cohort of clinicians who continuously practiced from 2018 to 2022 and treated at least 1 patient with OUD (*International Statistical Classification of Diseases and Related Health Problems, Tenth Revision*, code F11) annually. We defined tele-buprenorphine initiation as a patient’s first buprenorphine prescription (after a ≥90-day period with no buprenorphine prescriptions) with a tele-OUD visit 7 days before or 3 days after the prescription (eAppendix in Supplement 1).^3^ We measured the proportion of tele-buprenorphine initiations between March 16, 2020 (start of temporary telehealth rules), and November 30, 2022 (allowing measurement of 30-day follow-up through December 2022), with (1) no in-person visit with the tele-initiating clinician within 2 years prior to initiation (reflecting initiations not allowed under pre–COVID-19 pandemic rules) and (2) neither 2-year preinitiation nor 30-day follow-up in-person visits with the initiating clinician (reflecting proposed post–COVID-19 pandemic rules). We stratified by year, prescribing clinician type, and patient insurance type, and calculated 95% CIs using Stata/SE version 16.0 (StataCorp). The study was approved by the Johns Hopkins Bloomberg School of Public Health institutional review board with a waiver of informed consent and followed the STROBE reporting guideline for cross-sectional studies.

## Results

**Figure 1.**
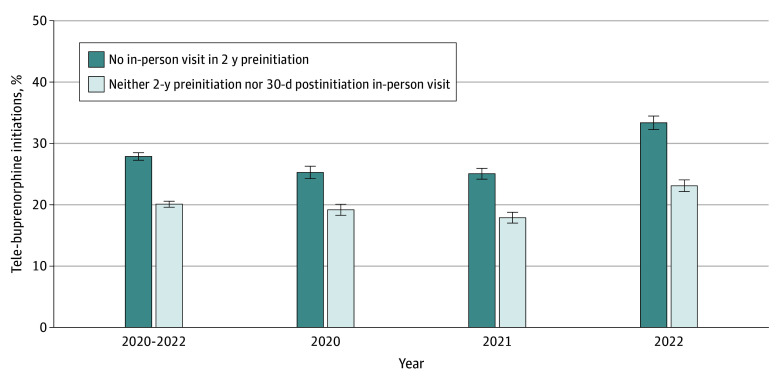
Tele-Buprenorphine Initiations Without In-Person Patient-Clinician Visits, 2020-2022 Data are from IQVIA medical and pharmaceutical claims. The figure presents the proportion of the 22 601 total buprenorphine initiations conducted via telehealth between March 16, 2020, and November 30, 2022, in which the patient had (1) no in-person visit with the initiating clinician in the 2 years prior to initiation (6313 overall from 2020-2022) and (2) neither 2-year preinitiation nor 30-day postinitiation in-person visits (4537 overall from 2020-2022). The error bars indicate 95% CIs.

**Figure 2.**
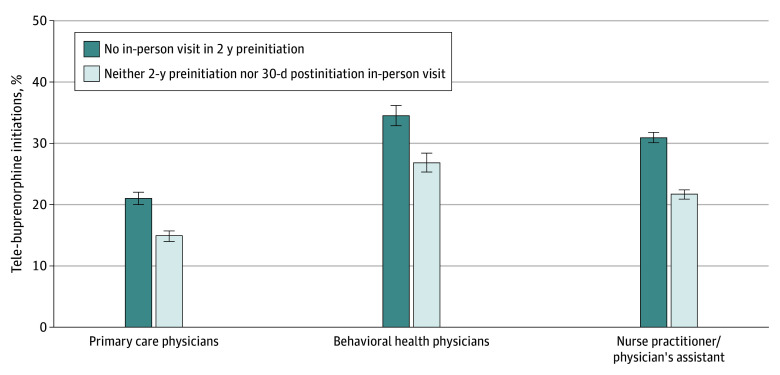
Tele-Buprenorphine Initiations Without In-Person Patient-Clinician Visits by Clinician Type, 2020-2022 Data are from IQVIA medical and pharmaceutical claims. The figure presents the proportion of total buprenorphine initiations conducted by primary care physicians (1412 physicians and 6722 initiations), behavioral health physicians (660 physicians and 3729 initiations), and nurse practitioners or physician’s assistants (1569 clinicians and 11 114 initiations) via telehealth between March 16, 2020, and November 30, 2022, in which the patient had (1) no in-person visit with the initiating clinician in the 2 years prior to initiation and (2) neither 2-year preinitiation nor 30-day postinitiation in-person visits. The error bars indicate 95% CIs. Primary care physicians were defined as physicians specializing in internal and family medicine. Behavioral health physicians were defined as physicians specializing in psychiatry, addiction medicine, addiction psychiatry, child and adolescent psychiatry, geriatric psychiatry, psychiatry neurology, pediatric psychiatry, forensic psychiatry, and psychiatry family medicine.

Of 228 598 total buprenorphine initiations between March 2020 and November 2022, 22 601 initiations (9.9%) by 3950 clinicians to 21 220 patients were via telemedicine. Among tele-initiations, 6313 (27.9%; 95% CI, 27.3%-28.5%) had no in-person visit with the initiating clinician in the prior 2 years, and 4537 (20.1%; 95% CI, 19.6%-20.6%) had neither 2-year prior nor 30-day follow-up in-person visits with the initiating clinician (Figure 1); the remainder had in-person visits in these time frames. The proportions of tele-initiations with neither 2-year prior nor 30-day follow-up in-person visits with the initiating clinician were 19.2% (95% CI, 18.3%-20.1%) in 2020, 17.9% (95% CI, 17.0%-18.8%) in 2021, and 23.1% (95% CI, 22.2%-24.1%) in 2022. Overall, 14.9% (95% CI, 14.0%-15.7%) of tele-initiations by primary care physicians (PCPs), 26.8% (95% CI, 25.3%-28.4%) by behavioral health physicians, and 21.7% (95% CI, 20.9%-22.4%) by nurse practitioners or physician assistants (NPs/PAs) had neither 2-year prior nor 30-day follow-up in-person visits (Figure 2). Among initiations for Medicaid, Medicare, and commercial beneficiaries, 16.3% (95% CI, 15.6%-17.0%), 27.6% (95% CI, 25.7%-29.6%), and 26.4% (95% CI, 24.6%-27.2%), respectively, had no in-person visits in these time frames.

## Discussion

The proposed DEA rule could impede buprenorphine initiation for a substantial number of patients with OUD. In our study, more than 4500 tele-buprenorphine initiations from 2020 to 2022—20% of tele-initiations overall—would have been prohibited under requirements for an in-person visit with the tele-initiating clinician prior to or within 30 days after tele-initiation. Behavioral health physicians and NPs/PAs treating patients with OUD, who conducted approximately one-quarter of tele-initiations from 2020 to 2022 without these in-person visits, could be particularly affected relative to PCPs, who conducted 15%. While no studies have compared the effectiveness of tele-initiations with vs without prior or follow-up in-person visits with the initiating clinician, tele-buprenorphine initiations overall have been associated with greater OUD treatment engagement and lower overdose rates than in-person initiations.^6^

Study findings are limited by lack of data from 2023 to 2024. In addition, some patients likely had in-person visits with buprenorphine-initiating clinicians outside the 2-year look-back period.

## Notes

Supplement 1. **eAppendix.** Telehealth Codes and Descriptions

Supplement 2.
Data Sharing Statement
