# Supplementary material for: Tele-Buprenorphine Initiations for Opioid Use Disorder Without In-Person Relationships
Source: JAMA Netw Open. 2025 Mar 3;8(3):e250001. doi: 10.1001/jamanetworkopen.2025.0001 (PMC11877218; doi:10.1001/jamanetworkopen.2025.0001)
Supplement: Supplement 1. — eAppendix. Telehealth Codes and Descriptions [file jamanetwopen-e250001-s001.pdf]

Supplemental Online Content

McGinty EE, Ge Y, Yu J, Tormohlen KN, Eisenberg MD. Tele-buprenorphine initiations for opioid use disorder without in-person relationships. *JAMA Netw Open.* 2025;8(3):e250001. doi:10.1001/jamanetworkopen.2025.0001

eAppendix. Telehealth Codes and Descriptions

This supplemental material has been provided by the authors to give readers additional information about their work.

© 2025 McGinty EE et al. *JAMA Network Open*.

eAppendix. Telehealth Codes and Descriptions

Type	CPT Code	Description
Telehealth facility claim	Q3014	Telehealth facility fee
Telehealth facility claim	T1014	For telehealth transmission, per minute
Telehealth, inpatient	G0406	Follow-up inpatient telehealth, limited, 15 min
Telehealth, inpatient	G0407	Follow-up inpatient telehealth, intermediate, 25 min
Telehealth, inpatient	G0408	Follow-up inpatient telehealth, complex, 35 min
Telehealth, inpatient	G0425	Initial inpatient/ED telehealth, 30 mins
Telehealth, inpatient	G0426	Initial inpatient/ED telehealth, 50 mins
Telehealth, inpatient	G0427	Initial inpatient/ED telehealth, 70+ mins
Telehealth, inpatient	G0459	Inpatient pharmacologic management
Telehealth, critical care	G0508	Initial critical care telehealth consult, 60 min
Telehealth, critical care	G0509	Subsequent critical care telehealth consult, typically 50 min
Telephone	G2025	RHC/FQHC distant site telehealth service
Telephone	99441	Non-Face-to-Face Telephone E&M Services, 5-10 mins
Telephone	99442	Non-Face-to-Face Telephone E&M Services, 11-20 mins
Telephone	99443	Non-Face-to-Face Telephone E&M Services, 21-30 mins
Telephone	98966	A nonphysician provider telephone E&M services, 5-10 mins
Telephone	98967	A nonphysician provider telephone E&M services, 11-20 mins
Telephone	98968	A nonphysician provider telephone E&M services, 21-30 mins
Online E&M	99421	Non-Face-to-Face On-Line Digital E&M Service, 5-10 mins
Online E&M	99422	Non-Face-to-Face On-Line Digital E&M Service, 11-20 mins
Online E&M	99423	Non-Face-to-Face On-Line Digital E&M Service, 21+ mins
Online E&M	G2061	Online Assessment of established patient by Qualified Nonphysician Healthcare Professional, 5-10 mins
Online E&M	G2062	Online Assessment of established patient by Qualified Nonphysician Healthcare Professional, 11-20 mins
Online E&M	G2063	Online Assessment of established patient by Qualified Nonphysician Healthcare Professional, 21+ mins
Online E&M	98970	replaced code G2061 in 2021
Online E&M	98971	replaced code G2062 in 2021
Online E&M	98972	replaced code G2063 in 2021
Online check-in	G2012	Brief communication technology-based service, e.g. virtual check-in, to an established patient
Online check-in	G2251	Brief communication technology-based service by a qualified health care professional who cannot report evaluation and management services, 5-10 mins
Online check-in	G2252	Brief communication technology-based service by a physician or other qualified health care professional who can report evaluation and management services, 11-20 mins
**Type**	**CPT Code Modifiers**	

© 2025 McGinty EE et al. *JAMA Network Open*.

Telephone, modifier	93	Modifier for audio-only telemedicine
Telehealth, modifier	GT	Modifier code for synchronous telemedicine
Telehealth, modifier	GQ	Modifier code for asynchronous telemedicine
Telehealth, modifier	95	Modifier code for synchronous telemedicine
Telehealth, modifier	G0	Modifier code for synchronous telemedicine for acute stroke
Mental health telehealth, modifier	FR	Modifier code for supervising practitioner present during synchronous telemedicine for mental health services
Mental health telephone, modifier	FQ	Modifier code for audio-only telemedicine for mental health services

Insurance Categories

Analyses stratified results for tele-buprenorphine initiations among patients insured by Medicare, Medicaid, or commercial insurance, the three largest insurance groups. This analysis excluded tele-initiations among patients insured by other payers including integrated plans for dual Medicare/Medicaid eligible individuals, state employee health plans, and Veteran's Health Administration and uniformed services (TRICARE) insurance.

© 2025 McGinty EE et al. *JAMA Network Open*.
