# Supplementary material for: Tele-Buprenorphine Initiations for Opioid Use Disorder Without In-Person Relationships
Source: JAMA Netw Open. 2025 Mar 3;8(3):e250001. doi: 10.1001/jamanetworkopen.2025.0001 (PMC11877218; doi:10.1001/jamanetworkopen.2025.0001)
Supplement: Supplement 2. — Data Sharing Statement [file jamanetwopen-e250001-s002.pdf]

Data Sharing Statement

McGinty. Tele-Buprenorphine Initiations for Opioid Use Disorder Without In-Person Relationships. *JAMA Netw Open*. Published March 03, 2025.
doi:10.1001/jamanetworkopen.2025.0001

Data
Data available: No

Additional Information
Explanation for why data not available: The terms of the data licensing agreement between IQVIA and the Johns Hopkins Bloomberg School of Public Health do not allow us to share the data or data dictionary.
